# Hypotensive Effects and Angiotensin-Converting Enzyme Inhibitory Peptides of Reishi (*Ganoderma lingzhi*) Auto-Digested Extract

**DOI:** 10.3390/molecules190913473

**Published:** 2014-08-29

**Authors:** Hai-Bang Tran, Atsushi Yamamoto, Sayaka Matsumoto, Hisatomi Ito, Kentaro Igami, Toshitsugu Miyazaki, Ryuichiro Kondo, Kuniyoshi Shimizu

**Affiliations:** 1 Faculty of Agriculture, Kyushu University, Fukuoka 812-8581, Japan; E-Mails: haibangtran@gmail.com (H.-B.T.); atsushi.yamamoto0510@gmail.com (A.Y.); syk.matsumoto@gmail.com (S.M.); ryukondo@agr.kyushu-u.ac.jp (R.K.); 2 Beauty Care Products Division, Nagase & Co. Ltd., Nishi-ku, Kobe, 651-2241, Japan; E-Mails: hisatomi.ito@nagase.co.jp (H.I.); kentaro.igami@nagase.co.jp (K.I.); toshitsugu.miyazaki@nagase.co.jp (T.M.)

**Keywords:** ACE inhibitors, hypotensive effects, *de novo* sequencing, active peptides, auto-digested, Ganodermataceae, *Ganoderma lingzhi*

## Abstract

Reishi (*Ganoderma lingzhi*) has been used as a traditional medicine for millennia. However, relatively little is known about this mushroom’s proteins and their bioactivities. In this study, we used reishi’s own proteases to hydrolyze its protein and obtained auto-digested reishi (ADR) extract. The extract was subjected to *in vitro* assays and administered to spontaneous hypertensive rats (SHRs) to determine its potential for use as a hypotensive medication. Bioassay-guided fractionation and *de novo* sequencing were used for identifying the active compounds. After 4 h administration of ADR, the systolic pressure of SHRs significantly decreased to 34.3 mmHg (19.5% change) and the effect was maintained up to 8 h of administration, with the decrease reaching as low as 26.8 mmHg (15% reduction–compare to base line a decrease of 26.8 mmHg is less than a decrease of 34.3 mmHg so it should give a smaller % reduction). Eleven peptides were identified and four of them showed potent inhibition against ACE with IC_50_ values ranging from 73.1 μM to 162.7 μM. The results showed that ADR could be a good source of hypotensive peptides that could be used for antihypertensive medication or incorporation into functional foods.

## 1. Introduction

The medicinal values of the reishi mushroom (*lingzhi* in Chinese) were documented more than 2,000 years ago [1]. Nowadays, reishi’s pharmacological effects are the subject of renewed interest among researchers. A recent study reported by Cao *et al.* [2] indicated that the reishi commercially cultivated in East Asia, formally known as *Ganoderma lucidum*, is a different species from the true “*Ganoderma lucidum*” which was originally described as being from Europe. Cao *et al.* proposed the name “*Ganoderma lingzhi* Sheng H. Wu, Y. Cao & Y.C. Dai” for the reishi distributed in East Asia.

Containing approximately 400 different bioactive compounds, many of which have been found to be unique to this fungus, reishi has been reported to have effects on many kinds of diseases [1]. Besides triterpenoids and polysaccharides, which have been isolated and thoroughly investigated [3], some proteins and lectins have also been identified in reishi [4,5]. Previous studies have also provided valuable information on the existence of bioactive peptides in reishi [6,7]. Unlike other mushrooms, which have been considered rich sources of proteins, reishi contains only around 7%–8% of these macromolecules [8]. However, studies on reishi have indicated that the mushroom could be a good source for bioactive proteins and peptides used in the treatment of a variety of diseases.

Angiotensin-Converting Enzyme (ACE) plays an important physiological role in regulating blood pressure. It converts angiotensin I to angiotensin II, which constricts the blood vessels and therefore increases blood pressure. Inhibition of the enzyme remains one of the first-line options for treatment of hypertension [9]. Since the first ACE-inhibitory peptide was discovered in viper’s venom, there has been an ongoing search for natural ACE-inhibitory peptides, especially from food-derived proteins [10,11,12], and mushrooms [13,14,15,16]. Although proteolytic peptides are less potent (IC_50_ values in μM range) than synthetic ACE inhibitors (IC_50_ values in nM range), they have a potential as active components in the diet by integration into functional food products.

Currently, studies of ACE-inhibitory peptides have focused mainly on the production and characterization of peptides isolated from microbial fermentation or digestions of proteins by enzymes supplied from outside [17]. Relatively little is known about auto-digested peptides formed by the “inside enzymes”. In this study, we present for the first time the hypotensive effects of an auto-digested reishi extract (ADR) and the existence of ACE-inhibitory peptides in this extract. The results of this study will contribute to the development of functional foods or antihypertensive medication using reishi as a source of the bioactive compounds, as well as to the understanding and use of auto-digested products.

## 2. Results and Discussion

### 2.1. Reishi Proteases’ Activities

**Figure 1:**
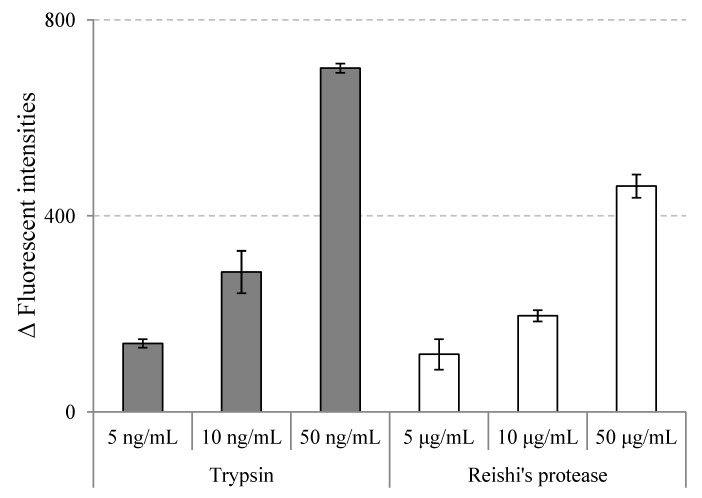
Proteolytic activities of reishi’s proteases extract at different concentrations; trypsin and TBS buffer were used as positive and negative controls, respectively; the activities were defined as the difference of fluorescent intensity between sample solutions and the negative control (value = mean ± SD, n = 3).

In this study, reishi was extracted with ion-exchange water for 4 h at 4 °C. This reishi extract was then centrifuged, lyophilized and applied to a protease assay kit to test for proteolytic activities. The results are shown in Figure 1.

A study on proteolytic activities of 43 mushrooms by Sabotic *et al.* [18] revealed a large variety of proteases from basidiomycetes mushrooms as in other kingdoms. The fact that many proteases from mushrooms showed distinctive characteristics, and could be exclusive to basidiomycetes, makes mushrooms a vast and potent source of novel proteases [19]. However, information on proteases of reishi is very limited. We could not find any report for proteases in *G.* *lingzhi*; and only two published studies reporting the metalloproteases of the *G. lucidum* mushroom could be found [20,21]. 

From the results presented in Figure 1, we can see that the proteolytic activity of reishi extract increased in a dose-dependent manner. This is the first time the total protease activity of reishi extract has been reported, and the results should provide useful information for future studies on reishi’s proteases. After reishi’s proteolytic activity was confirmed, reishi powder was subjected to an auto-digestion process and the resulting extract (ADR) was used for further investigation of *in vitro* ACE inhibition and *in vivo* hypotensive effects. Reishi hot water extract (HWR) was also used for comparison.

### 2.2. ACE Inhibition of Auto-Digested Reishi Extract

**Figure 2:**
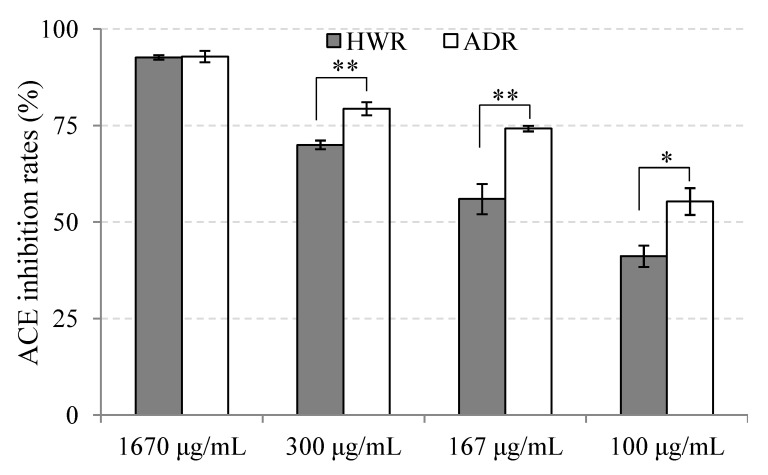
ACE inhibition rate of hot-water and auto-digested extracts of reishi at different concentrations (value = mean ± SD, n = 3). significant differences were determined by *t*-test (unpaired; one-tailed): *p* < 0.005 (*), *p* < 0.001 (**).

As shown in Figure 2, in all of the tested concentrations, both ADR and HWR showed ACE inhibition potential but ADR exhibited higher inhibitory activity than HWR. At a concentration as low as 100 μg/mL, ADR showed more than 50% inhibition. The inhibition rate increased to 75% at 167 μg/mL, and the enzyme was nearly completely inhibited at 1670 μg/mL. HWR also showed a dose-dependent inhibitory effect. The extract exhibited the same inhibition rate as that of ADR at 1670 μg/mL, even though it showed lower inhibition rates at other investigated concentrations. The difference of ACE inhibitory activity between ADR and HWR might be a consequence of the method of preparation. The auto-digestion process may have created active short peptides by proteolytic action of the mushroom’s own proteases while hot-water-extraction process did not. These peptides might be the main factors causing the observed increase in the inhibitory effect of the ADR.

ACE inhibitory activity has been reported for many mushroom extracts. However, due to the differences in the assay systems used for evaluation of ACE-inhibitory activity, it is difficult to compare the results of different studies. Assuming that IC_50_ values could reflect the inhibitory strength, we can partly compare the inhibitory capacity of samples reported in different studies. In this context, the ACE-inhibitory activity of ADR reported in this study exceeded those reported for water extracts of other mushrooms. In a study carried out by Ukawa [22] on nine mushrooms, cold-water extract of *Lyophyllum decastes* was reported to have the highest inhibition against ACE, but the IC_50_ value was as high as 250 μg/mL. In another survey on 23 mushrooms (screened from among 500 mushrooms) done by Izawa and Aoyagi [23], the hot-water extracts of 22 mushrooms showed lower IC_50_ values, ranging from 110–2250 μg/mL, and only one sample, *Pholiota adiposa*’s extract, gave a comparable result to that of ADR, with an IC_50_ value of 66 μg/mL. ADR also showed better inhibitory activities than most mushrooms’ water extracts of a recent study on antihypertensive proteins of nine edible mushrooms reported by Lau *et al.* [24]. At a concentration of 1670 μg/mL, ADR inhibited up to 93% ACE activity; however, most of the mushrooms’ extract in Lau’s experiment required concentrations as high as 10,000 μg/mL to reach this rate. Based on these comparisons, the ADR of this study certainly merits further investigation of its *in vivo* antihypertensive effect.

### 2.3. Hypotensive Effects of Reishi and Auto-Digested Reishi Extract on Rats

**Figure 3:**
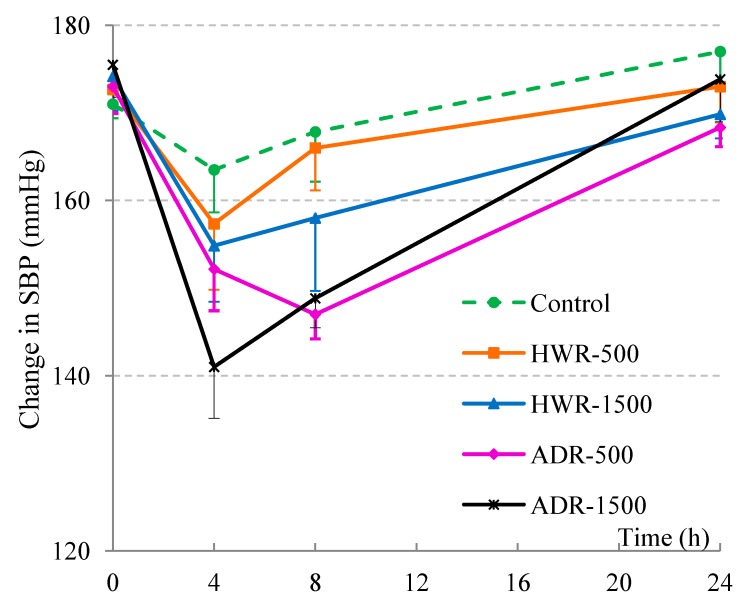
Time-course of changes in SBP of SHRs after administering HWR and ADR extracts (average ± SE, n = 6). Single oral administration was dosed at 500 and 1500 mg/kg body weight. Ultrapure water was used as control. Two-way repeated measures ANOVA (Group × Time), with “H” as the within-subjects variable and “Group” as a between-subjects variable followed by a simple main effect test was applied. There was no significant Group effects (*F*(4,25) = 1.93, *p* = 0.136), but significant Time (*F*(3,75) = 32.43, *p* < 0.001) and Group × Time interaction effect (*F*(12,75) = 2.08, *p* = 0.028) were observed.

Reishi has a long history of use for promoting health and has been believed to have many therapeutic properties including strengthening of the cardiovascular function. Reishi powder was reported to have hypotensive effects on spontaneously hypertensive rats [25] and hot-water extract of reishi showed ameliorating effects on essential hypertensive patients after six months’ treatment [26]. In this study ADR and HWR were administered to spontaneous hypertensive rats (SHRs) at doses of 500 and 1500 mg/kg bodyweight, respectively. The initial average blood pressures of the SHRs in the test group were in the range of 171‒175 mmHg just before the experiments. Systolic blood pressure (SBP) was measured at 0 and 4, 8, and 24 h after administration (time points denoted as T_0_, T_4_, T_8_ and T_24_, respectively) and the results are shown in Figure 3.

As shown in Figure 3, at T_0_, there was no difference among groups in both systolic and diastolic blood pressure (for diastolic blood pressure, DBP, see Supplementary Materials, Figure S1). However, after 4 h, both reishi-treated groups showed low systolic blood pressure (SBP); after 8 h, three groups including ADR-500, ADR-1500 and HWR-1500 were still at a low level of SBP. After 24 h, all groups’ SBPs had returned to baseline levels. There was also a tendency of lowering of DBP from T_0_ to T_4_, but no difference between T_4_ and T_8_ was observed, and all groups’ DBP had returned to starting levels at 24 h. To confirm the variations, SBP was subjected to repeated analysis of variance (five groups) × time (four points) followed by a simple main effect test. There was no significant Group effect (*F*(4,25) = 1.93, *p* = 0.136), but significant Time (*F*(3,75) = 32.43, *p* < 0.001) and Group × Time interaction effects (*F*(12,75) = 2.08, *p* = 0.028) were observed. A simple main effect test also revealed a significant difference at T_8_ (*F*(4,25) = 3.16, *p* = 0.031) but not at other time points. There was no observable difference for the control group (*F*(3,23) = 2.26, *p* = 0.116), but the simple main effect test for each group at all time-points showed significant differences in all sample-administered groups (HWR-500 group’s *F*(3, 23) = 4.15, *p* = 0.017; HWR-1500 group’s *F*(3, 23) = 8.29, *p* = 0.001; ADR-500 group’s *F*(3, 23) = 14.95, *p* < 0.001; ADR-1500 group’s (*F*(3, 23) = 27.66, *p* < 0.001).

Four hours after administration of the extracts, SBP decreased in all test groups; the sharpest decrease was observed in the ADR-1500 group with the change of SBP reaching −34.3 mmHg (or 19.5% of change). Interestingly, 8 h after administration of the extract, while other groups showed a tendency to lose their hypotensive effect, the ADR-500 group maintained its effect with an activity even higher than that at 4 h and similar to that of ADR-1500 (15% of reduction). It is also worth noting that the water extracts of other mushrooms and their active compounds reported in previous studies [14,15,16] tend to lose their hypotensive effect only 4 h after administration. The fact that the hypotensive activity of ADR is still present after 8 h of administration may be due to the existence of intestinal digestion-resistant hypotensive agents in the extract. Base on the data presented here, ADR could be a promising candidate for use in antihypertension medication or for incorporation into antihypertensive functional foods.

### 2.4. Fractionation and Identification of ACE-Inhibitory Peptides

**Figure 4:**
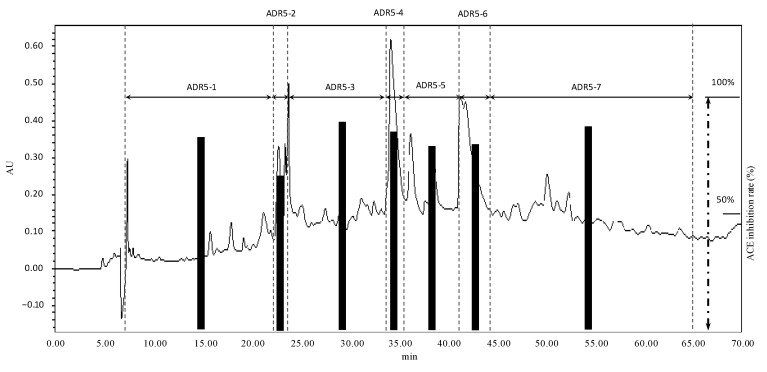
Typical chromatogram of RP-HPLC fractionation of the ADR-5 (solvent program was a linear gradient from 5%–13% of 0.1% TFA/AcCN in 0.1% TFA/H_2_O for 60 min; then increased to 25% in other 10 min before finished) and the sub-fractions’ ACE-inhibitory effects (100 μg/mL, n = 3; mean values and standard deviations were in Supplementary Materials, Table S2).

Presently, ACE inhibitors are the second most-commonly prescribed treatment for hypertension [27] and the popular assumption for hypotensive effect of peptides is due to this enzyme blockade [28]. Studies on ACE-inhibitory peptides have mainly focused on the ≤3-kDa fraction due to the limitation of the dimensions of the enzyme’s active site. In a study carried out by Kumakura [29], the author also proposed that the ≤3-kDa fraction of reishi extract may contain ACE-inhibitory peptides. The ADR extract of this study was proven to have *in vitro* ACE inhibition and *in vivo* hypotensive effect. Therefore, in tandem with previous studies’ results, we decided to make further investigation on the components of ADR to clarify the potent components causing the hypotensive effect on SHRs as reported above. ADR was subjected to ultrafiltration using a 3-kDa cut-off membrane and after confirming the existence of peptides and/or amino acid in the ≤3-kDa fraction by staining with ninhydrin (Supplementary Materials, Figure S2), the permeate was then subjected to RP-HPLC for further fractionation. A typical chromatogram of a semi-preparative RP-HPLC fractionation and the corresponding fractions’ ACE inhibition rates are shown in Figure 4.

After the first fractionation by HPLC, 11 fractions were obtained (see Supplementary Materials, Figure S3, Table S1), but only fraction ADR5, which had the highest inhibition rate and a sufficient quantity for further investigation, was chosen for further separation by a semi-preparative HPLC to obtain 7 other sub-fractions. From the results shown in Figure 4 we can see that at 100 μg/mL, most sub-fractions had inhibition rates of more than 80% and there were no great differences in ACE inhibition between fractions. These results suggested that the active peptides were distributed evenly in all fractions and there was no single dominant compound for the inhibition of ACE. From these results we decided to apply all fractions onto an LCMS system to identify the structures contributing to the inhibitory activities against ACE.

In order to identify the potent ACE inhibitory peptides, all fractions (ADR5-1–ADR5-7) were analyzed by LC-MS/MS. Firstly, the peptides candidates were identified by *de novo* sequencing. All the candidates were then chemically synthesized and applied to the LC-MS/MS system for checking the fragmentation models. Peptide sequences were only accepted if the candidate’s mass spectrum and synthesized peptide’s mass spectrum matched with each other (see Supplementary Materials, Figure S4). In total 11 peptides were recognized and all of them were checked for the ACE-inhibitory effect. The results are shown in Table 1.

**Table 1:** Peptides existing in ADR extract and their ACE inhibition capacity

Fractions	IC_50_ Values (μM)			
	Dipeptides		Tripeptides	
ADR5-1	undetectable			
ADR5-2	Ser-Ile	>200	undetectable	
	**Ala-Tyr**	**162.7**		
	**Ser-Tyr**	**94.7**		
ADR5-3	undetectable			
ADR5-4	Ser-Leu	>200	Asn-Ser-Ile	342.1
			Lys-Val-Pro	>500
ADR5-5	Ala-Leu	>200	undetectable	
	Thr-Leu	>200		
ADR5-6	**Ile-Arg**	**73.7**	**Ile-Pro-Thr**	**73.1**
ADR5-7	undetectable		Gly-Pro-Leu	>500
Positive control			Ile-Pro-Pro	< 0.5

This time, only di- and tripeptides could be found in all sub-fractions. Although several parts were unidentifiable, there was no evidence of oligopeptides with more than three residues (>500 Da) in these fractions. It can be seen that four (boldface) of the 11 peptides have rather high inhibition activities. The IC_50_ values of these peptides range from 73.1 μM to 162.7 μM (or 25.4 to 40.8 μg/mL). Surprisingly, even ADR5-2 showed the lowest inhibition rate among seven sub-fractions of ADR5; its peptides were amongst the four highest activity peptides identified. This phenomenon might be attributed to the effect of unidentifiable part of the fraction. It can be predicted that the unidentifiable part contains some inactive components mixing with the active components of ADR5-2 reducing the inhibition rate of the fraction. Assuming that IC_50_ values partly reflect the inhibitory strength, the ACE-inhibitory activity of peptides identified in this study exceeded those recognized from water extracts of *Grifola frondosa* [13], *Tricholoma giganteum* [14], *Pholiota adiposa* [15] and *Pleurotus cornucopiae* [16], but were surpassed by some peptides isolated from *Pleurotus cystidiosus* [30] and *Agaricus bisporus* [31].

Blood pressure is regulated by various factors involved in the renin-angiotensin-aldosterone system, the sympathetic nervous system, and the kidney and fluid balance mechanisms. One limitation of this study is that only ACE was targeted for the potential of hypotensive effect of ADR and the extract’s peptides. However, ACE is reported to play a crucial role in blood pressure regulation and fluid and electrolyte balance [32] and data of ACE-inhibitory activity should give valuable information for further antihypertensive investigation. Besides, this limitation did not change the fact that the auto-digested extract containing ACE-inhibitory peptides showed hypotensive effect *in vivo*.

## 3. Experimental Section

### 3.1. Materials

*Ganoderma lingzhi* (BMC 4049 strain) powder was obtained from the Beauty Care Products Division of Nagase & Co. (Kobe, Japan). Synthetic peptides (>95%) were purchased from Medical & Biological Laboratories Co., Ltd. (Nagoya, Japan). Positive control, Isoleucine-Proline-Proline (IPP, >95%) was from Phoenix Pharmaceuticals (Funakoshi, Tokyo, Japan). Colorimetric assay kits for ACE activity were purchased from Dojindo Corp. (Dojindo Laboratories, Kumamoto, Japan) while protease assay kit was from Thermo Scientific (Pittsburgh, PA, USA). Spontaneous hypertensive rats (SHRs) were purchased from Japan SLC (Tokyo, Japan). The standard diet for rats was obtained from Nosan Corporation (Yokohama, Japan). Other reagents were from Wako Pure Chemical Industries, Ltd. (Osaka, Japan) and were of the finest analytical grade.

### 3.2. Preparation of Reishi’s Protease Extract and Its Proteolytic Activities

Reishi powder was air-dried and extracted by ion-exchange water for 4 h at 4 °C using an orbital shaker. The resulting mixture was then centrifuged for 10 min at 10,000 rpm and the supernatant was decanted and freeze-dried. Lyophilized samples were subjected to the Pierce™ Fluorescent Protease Assay Kit (Pierce, Rockford, IL, USA) which uses fluorescence detection following proteolytic digestion of fluorescein isothiocyanate-labeled casein (FTC-casein) to test the protease activities of mushroom extract. Briefly, the procedures were as follows: 100 μL of FTC-casein working solution was incubated with 100 μL of varied concentrations of reishi protease extract (5, 10 and 50 μg/mL) in a 96-well plate at room temperature. After 60 min of incubation time, the fluorescence intensity (excitation/emission was 485/530 nm) was measured using CytoFluor II Microplate Reader (PerSeptive Biosystems, Framingham, MA, USA). TBS buffer (25 mM Tris, 150 mMNaCl, pH 7.2) and bovine pancreas TPCK trypsin (>14,000 BAEE units/mg, supplied with the kit) dissolved in TBS buffer were used as the negative control and positive control, respectively. The proteolytic activities were defined as the difference of fluorescent intensity between sample solutions and the negative control.

### 3.3. Preparation of Reishi and Auto-Digested Reishi’s Extracts

*Reishi hot-water extract (HWR):* Reishi powder (280 g) was boiled with ion-exchange water for 3 h; the sample mixture was then filtered, the resulting extract was lyophilized, and the final extract powder (15.3 grams) was stored in a capped plastic bottle sealed with Parafilm and stored at 4 °C until assayed.

*Auto-digested extract (ADR):* Totally, 12 liters of ion-exchange water was added to a tank containing 840 grams reishi powder, and the mixture was incubated at 37 °C for 24 h with shaking every 1 h. The whole mixture was then autoclaved at 121 °C for 15 min. After being filtered with filter paper, the resulting extract was lyophilized and the dried powder (58.9 gram) was stored in a capped plastic bottle sealed with parafilm and stored at 4 °C until assayed.

### 3.4. ACE Inhibition Assay

Dojindo ACE Kit-WST A502 was used for testing the ACE-inhibitory activity. Principles of the kit were described in details in a previous report [33]. Testing procedures were run strictly according to the manufacturer’s instructions using a 96-well plate, and inhibition rate was calculated based on the comparison of optical absorbance of sample-treated wells (As), control wells (Ac) and blank wells (Ab) as in the equation below. Absorbance was measured at 450 nm using microplate reader-Biotek-ELX800 (BioTek, Winooski, VT, USA):

Inhibition rate (%) = [(Ac − As)/(Ac − Ab)]×100
(1)

For HWR and ADR extracts, four concentrations (100, 167, 300 and 1670 μg/mL) were assayed against ACE to test for the ACE inhibitory potency of the extracts. For the peptides, six different concentrations of each peptide, ranging from 10 to 100 μg/mL, were assayed, and dose-response curves were plotted for the calculation of the IC_50_ values. IPP has been proven to be an ACE inhibitor [34] and thus was used as positive control (at concentrations ranging from 0.15 to 5 μg/mL).

### 3.5. Hypotensive Effects of HWR and ADR Extract on SHR Rats

Twelve-week old spontaneously hypertensive male rats, SHR/Izm (Japan SLC, Shizuoka, Japan), weighing 269 to 290 grams, were divided randomly and housed in polycarbonate cages (three rats per cage) in a room kept at 23 ± 2 °C with a 12-h light/dark cycle. Experimental animals were fed a standard diet *ad libitum* and tap water. After a 1-week adaptation period rats were administered orally with HWR and ADR solutions at doses of 500 and 1500 mg/kg body weight. A group in which ultrapure water (20 mL/kg body weight) was injected instead of reishi samples was used as control. Blood pressure was measured before as well as 4, 8 and 24 h after administration by the tail-cuff method using a BP-98A machine (Softron Corporation, Tokyo, Japan). At each time point, the rats’ blood pressures were measured three times and mean values were retained for each rat; totally 6 mean values (for 6 rats in each group) were used for calculation of mean of the group. All of the animal experiments in this study were conducted in compliance with the guidelines of the Japanese Association for Laboratory Animal Science (2007) and approved by Animal Experiments of the Research and Development Division of Kyushu University (Approved date: 2010/12/28; Permitted Number: A22-234-0).

### 3.6. Ultrafiltration and RP-HPLC for Fractionation

Auto-digested reishi extract was subjected to ultrafiltration using a 3-kDa cut-off membrane to obtain 02 fractions having molecular weights in the ranges >3 kDa and ≤3 kDa. The latter was applied to preparative HPLC (Series 600 HPLC, Waters, Milford, MA, USA) for 2 further fractionation steps. For the first preparative HPLC, an Inertsil ODS-3 (20 × 250 mm, 5μm) column was used while an Eclipse-XDB C_18_, (9.4 × 250 mm, 5μm) column was selected for the second step. Water-A and acetonitrile-B (both were in mixture with TFA at the concentration of 0.1%) were used as the mobile phase, and the absorbance of the eluent was monitored at 215 nm in both steps. In the first step, elution was performed with a linear gradient of B in A from 10% to 60% for 60 min at a flow rate of 5 mL/min; in the second step the solvent program was started at 5 percent of B and the separation process was executed by a linear increase of B up to 13% over 60 min, and then from 60 to 70 min, B was increased to 25%; the process was finished by a washing step with 25% of B in 20 min without samples collection. The flow rate of this step was 2 mL/min. All of the collected fractions were freeze-dried, kept in capped glass vials and stored in a refrigerator until assayed.

### 3.7. Identification of Peptides by LC-MS/MS

Identification of peptides was performed on a LCMS-IT-TOF system (Shimadzu, Kyoto, Japan) equipped with an electrospray ionization source. First, samples were separated by HPLC equipped with a ZIC-HILIC column (1.0 × 150 mm, Merck KGaA, Darmstadt, Germany). Then, the eluate from the HPLC was directly injected to an octopole ion-trap/time-of-flight system. For HPLC separation, all fractions (concentrations were 0.1 or 0.3 mg/mL) underwent a linear gradient elution, from 10% to 90% water (1% CH_3_COOH) in a mixture with acetonitrile (1% CH_3_COOH) for 45 min at a flow rate of 0.1 mL/min. Mass analysis was set up to run in both positive and negative modes in which mass spectra were acquired over the range of 57–1000 *m/z*. Molecular mass was determined by the singly charged (M + H)^+1^ state in the mass spectrum. The peptide molecular mass was automatically selected for fragmentation, and sequence information was obtained from tandem MS analysis.

### 3.8. Statistical Analysis

All ACE-inhibitory assays were performed in triplicate whereas hypotensive test were repeated in 6 different rats for each group. significant differences of *in vitro* assays were determined by Student’s *t*-tests (unpaired; one-tailed) while two-way repeated measures ANOVA (Group × Time), with “H” as the within-subjects variable and “Group” as the between-subjects variable followed by a simple main effect test was applied for animal experiments. Values of *p* < 0.05 were considered to indicate statistical significance.

## 4. Conclusions

In this study, we investigated the *in vitro* ACE-inhibitory activity and *in vivo* hypotensive effect of auto-digested reishi in comparison with hot-water extract of the mushroom. Components showing potent activity against angiotensin-converting enzyme were also studied. It was shown that auto-digested reishi extract had higher inhibitory effect against ACE than that of the hot-water extract. The *in vitro* ACE assay suggested that no single ACE inhibitor dominated in the extract, but rather an aggregate of active peptides was present. Eleven peptides were identified and their IC_50_ values against ACE confirmed the hypothesis that a series of strong ACE inhibitors exists in auto-digested reishi extract. The *in vivo* hypotensive effect of auto-digested reishi extract suggested it to be a good source of ACE-inhibitory peptides that could be used in functional food or for antihypertension medication. This study not only reveals the remarkable properties of auto-digested reishi but also suggests a potentially more potent alternative to the water extracts of mushrooms, *i.e.*, auto-digested extracts.

## Supplementary Materials

Supplementary materials can be accessed at: http://www.mdpi.com/1420-3049/19/9/13473/s1.

## Supplementary Files

Supplementary File 1
