# Supplementary material for: Hypotensive Effects and Angiotensin-Converting Enzyme Inhibitory Peptides of Reishi (Ganoderma lingzhi) Auto-Digested Extract
Source: Molecules. 2014 Aug 29;19(9):13473–85. doi: 10.3390/molecules190913473 (PMC6271714; doi:10.3390/molecules190913473)
Supplement: Supplementary File 1 [file molecules-19-13473-s001.pdf]

Supplementary Materials

Figure S1. Time-course of changes (mean ± SE, n = 6) in DBP of SHRs after administering HWR and ADR extracts. Single oral administration was dosed at 500 and 1500 mg/kg body weight. Ultrapure water was used as the control (20 mL/kg body weight).

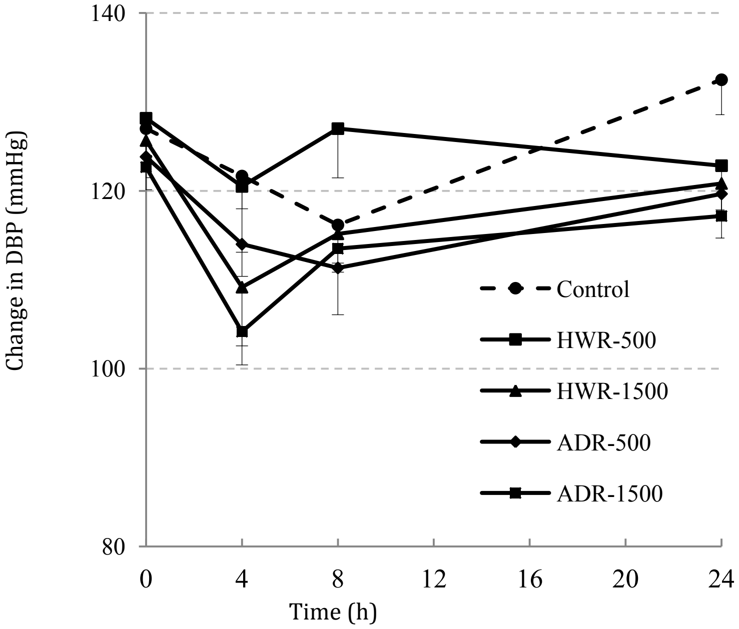

Figure S2. Ninhydrin staining of peptides and/or amino acid in the ≤3 kDa fraction of ADR extract.

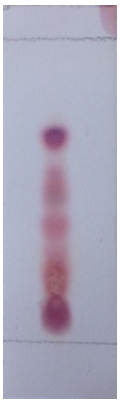

Reishi's auto-digestion extract was spotted onto silica-gel plate (TLC Plate Silica Gel 60 F254, Merck, Darmstadt, Germany); the plate was eluted with *n*-butanol:acetic acid:water 3:1:1 (by volume) mixture followed by ninhydrin stain (Wako Pure Chemical, Osaka, Japan)

Figure S3. Preparative HPLC chromatogram of the ≤3 kDa fraction. Elution was performed with a linear gradient from 10% to 60% of B for 60 minutes at a flow rate of 5 mL/min.

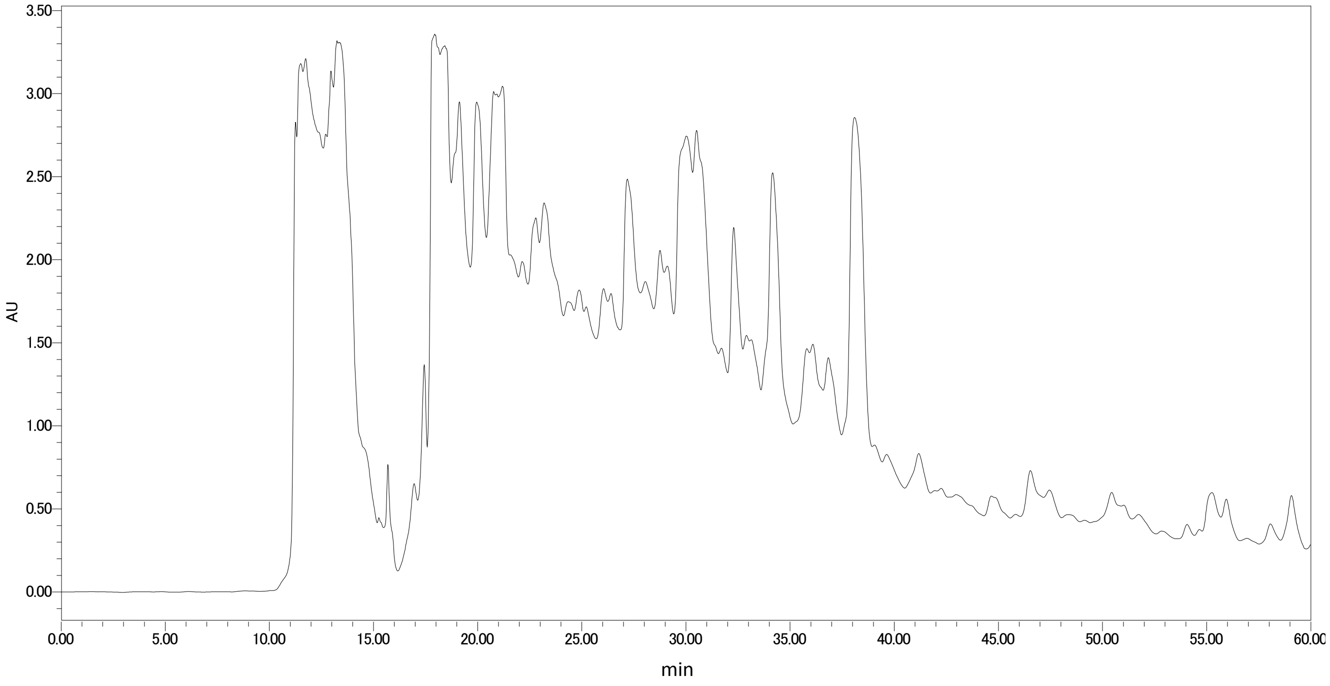

Figure S4. Mass spectra of identified peptide-candidates and corresponding standards.

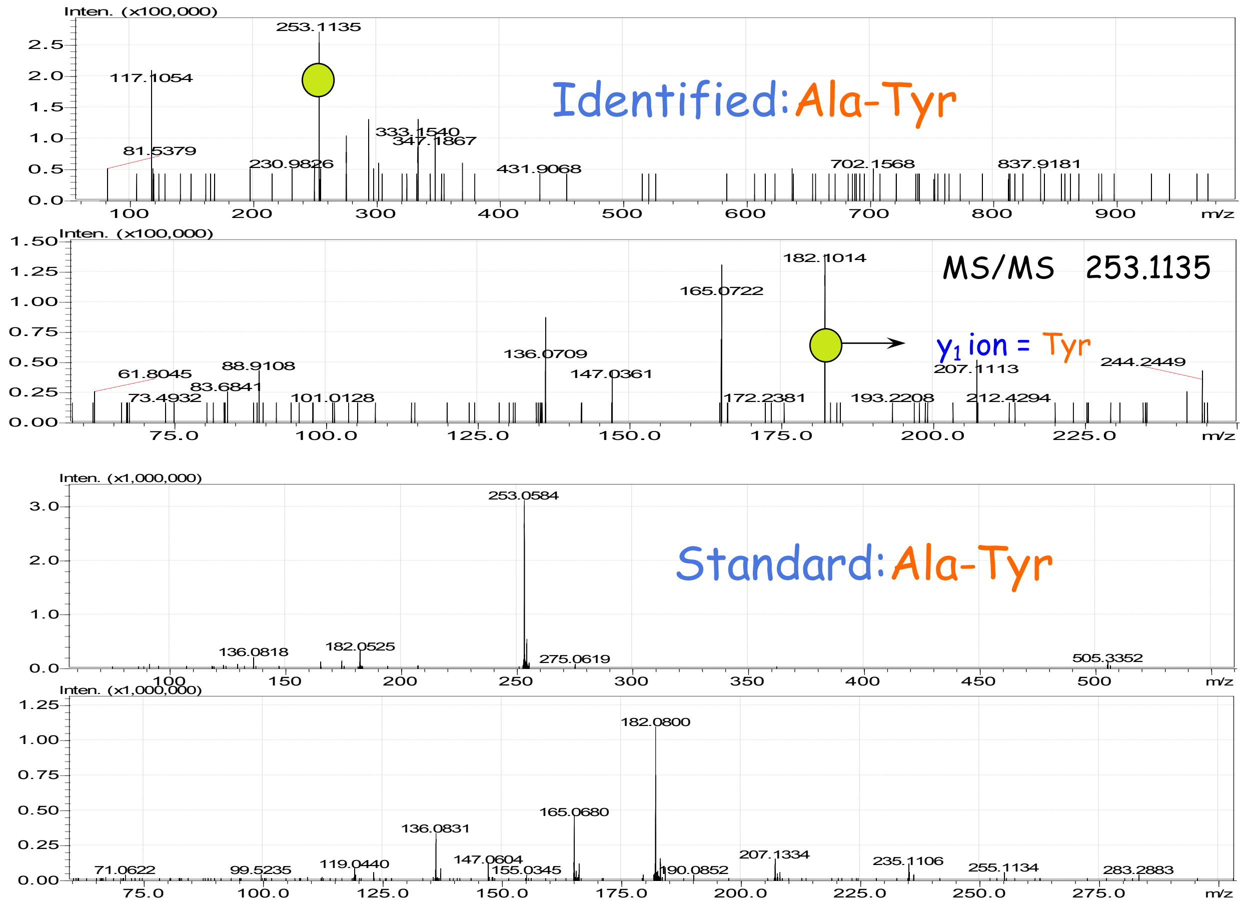

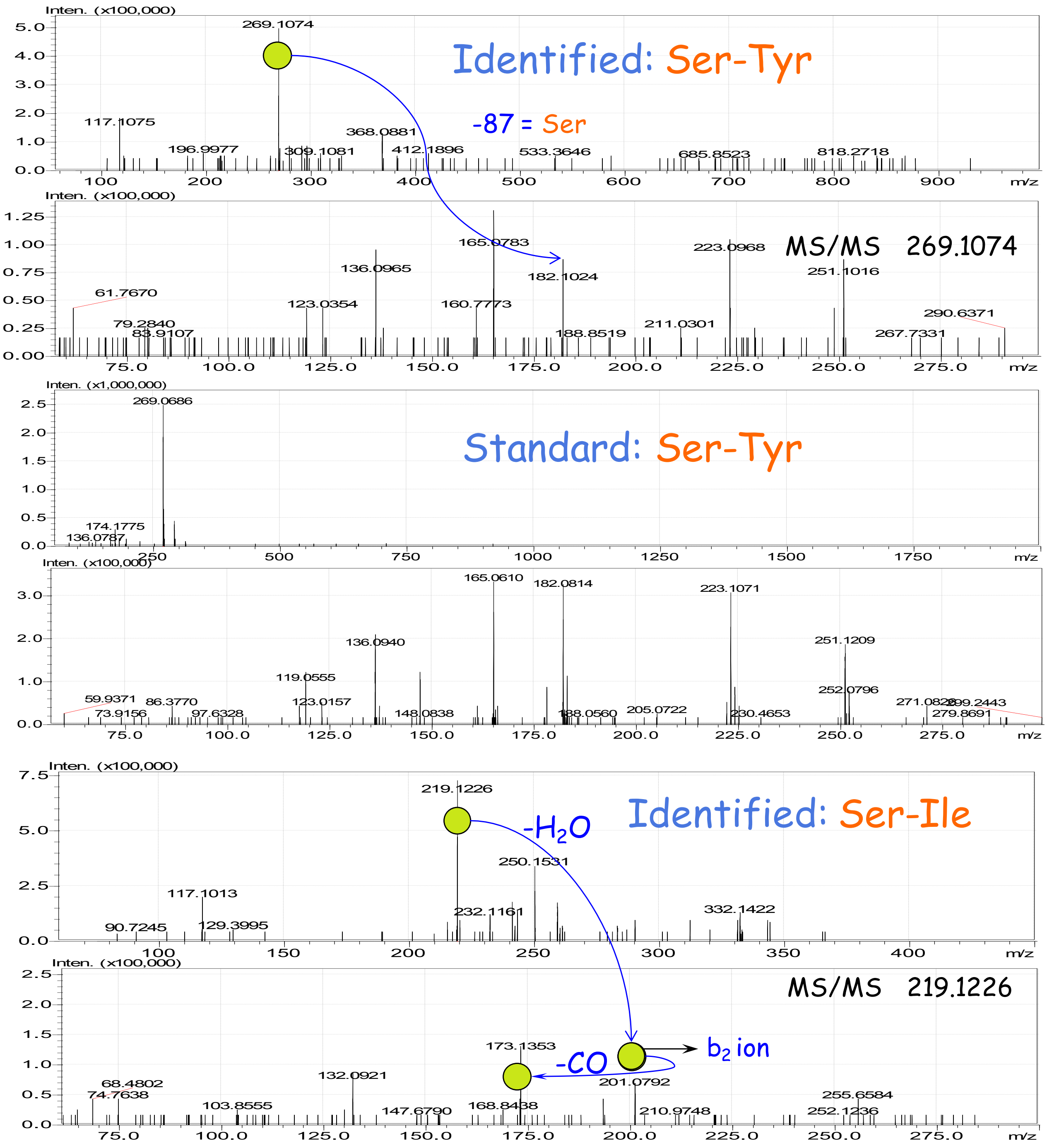

Inten. (x100,000)
269.1074
Identified: Ser-Tyr
-87 = Ser
117.1075
196.9977
309.1081
368.0881
412.1896
533.3646
685.8523
818.2718
m/z
Inten. (x100,000)
MS/MS 269.1074
165.0783
136.0965
182.1024
223.0968
251.1016
61.7670
79.2840
83.9107
123.0354
160.7773
188.8519
211.0301
267.7331
290.6371
Inten. (x1,000,000)
269.0686
Standard: Ser-Tyr
174.1775
136.0787
Inten. (x100,000)
165.0610
182.0814
223.1071
136.0940
251.1209
119.0555
123.0157
59.9371
73.9156
86.3770
97.6328
148.0838
188.0560
205.0722
230.4653
252.0796
271.0828
299.2443
279.8691
Inten. (x100,000)
219.1226
-H2O
Identified: Ser-Ile
250.1531
117.1013
232.1161
332.1422
90.7245
129.3995
Inten. (x100,000)
MS/MS 219.1226
b2 ion
173.1353
-CO
132.0921
201.0792
68.4802
74.7638
103.8555
147.6790
168.8438
210.9748
255.6584
252.1236

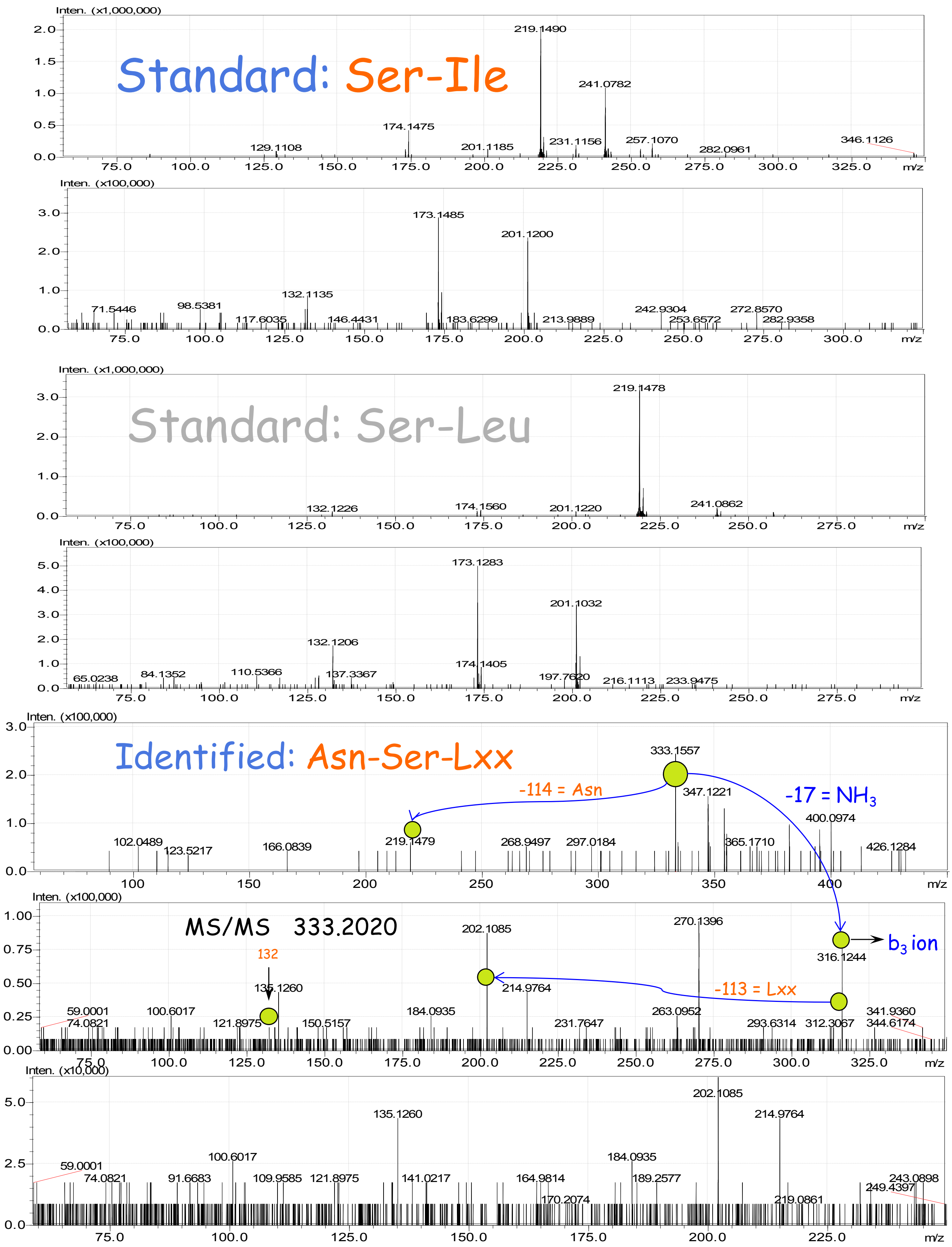

Standard: Ser-Ile
Inten. (x1,000,000)
219.1490
241.0782
174.1475
129.1108
201.1185
231.1156
257.1070
282.0961
346.1126
Inten. (x100,000)
173.1485
201.1200
71.5446
98.5381
132.1135
117.6035
146.4431
183.6299
213.9889
242.9304
253.6572
272.8570
282.9358
Standard: Ser-Leu
Inten. (x1,000,000)
219.1478
132.1226
174.1560
201.1220
241.0862
Inten. (x100,000)
173.1283
201.1032
132.1206
174.1405
65.0238
84.1352
110.5366
137.3367
197.7620
216.1113
233.9475
Identified: Asn-Ser-Lxx
Inten. (x100,000)
333.1557
-114 = Asn
-17 = NH3
347.1221
400.0974
102.0489
123.5217
166.0839
219.1479
268.9497
297.0184
365.1710
426.1284
Inten. (x100,000)
MS/MS 333.2020
270.1396
202.1085
132
135.1260
b3 ion
316.1244
214.9764
-113 = Lxx
59.0001
74.0821
100.6017
121.8975
150.5157
184.0935
231.7647
263.0952
293.6314
312.3067
341.9360
344.6174
Inten. (x10,000)
202.1085
135.1260
214.9764
100.6017
184.0935
59.0001
74.0821
91.6683
109.9585
121.8975
141.0217
164.9814
189.2577
170.2074
219.0861
243.0898
249.4397
m/z

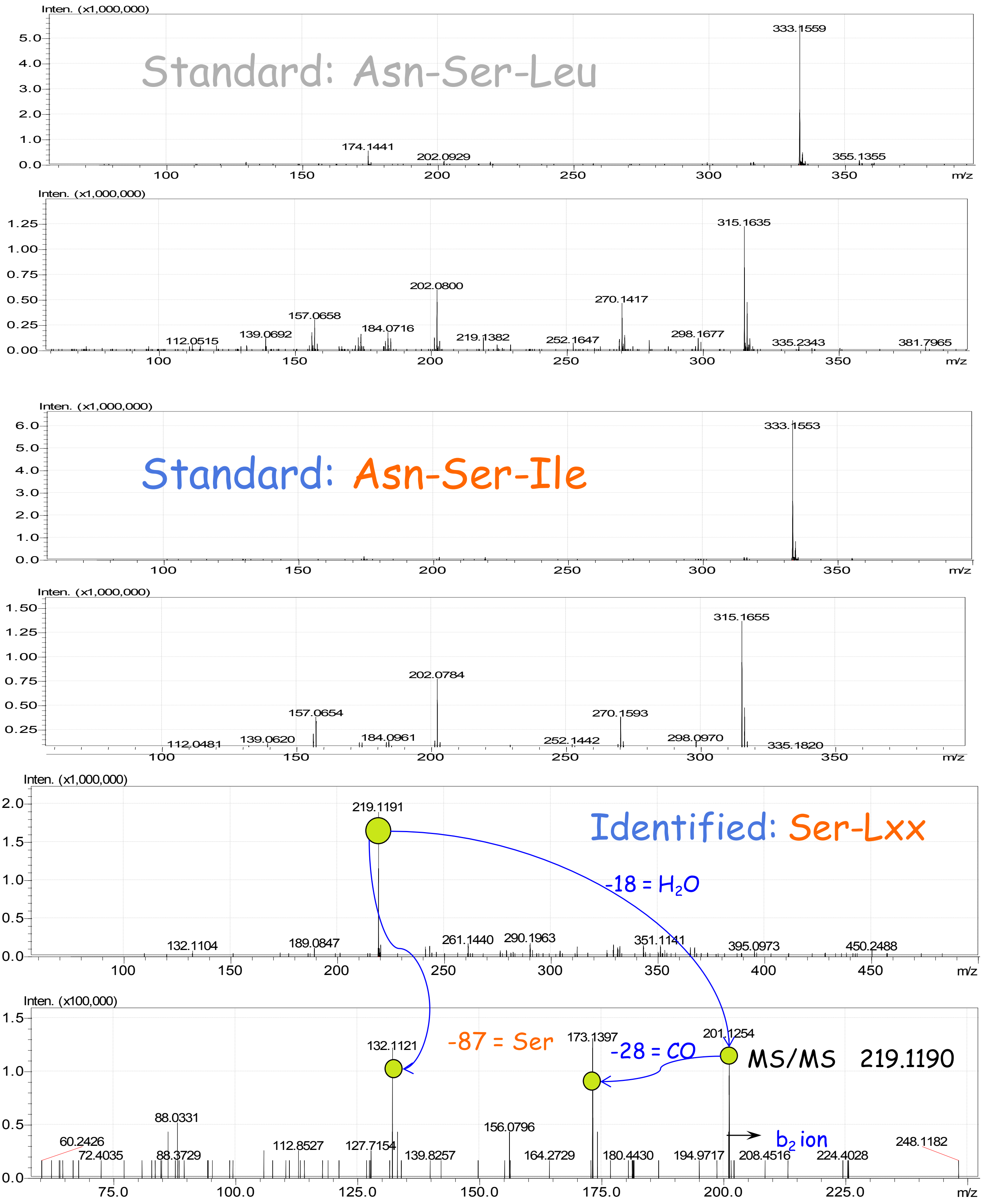
Standard: Asn-Ser-Leu
Inten. (x1,000,000)
333.1559
174.1441
202.0929
355.1355
315.1635
202.0800
270.1417
157.0658
184.0716
112.0515
139.0692
219.1382
252.1647
298.1677
335.2343
381.7965
Standard: Asn-Ser-Ile
333.1553
315.1655
202.0784
157.0654
270.1593
112.0481
139.0620
184.0961
252.1442
298.0970
335.1820
Identified: Ser-Lxx
219.1191
-18 = H2O
132.1104
189.0847
261.1440
290.1963
351.1141
395.0973
450.2488
Inten. (x100,000)
-87 = Ser
-28 = CO
MS/MS 219.1190
132.1121
173.1397
201.1254
b2 ion
60.2426
72.4035
88.0331
88.3729
112.8527
127.7154
139.8257
156.0796
164.2729
180.4430
194.9717
208.4516
224.4028
248.1182
m/z

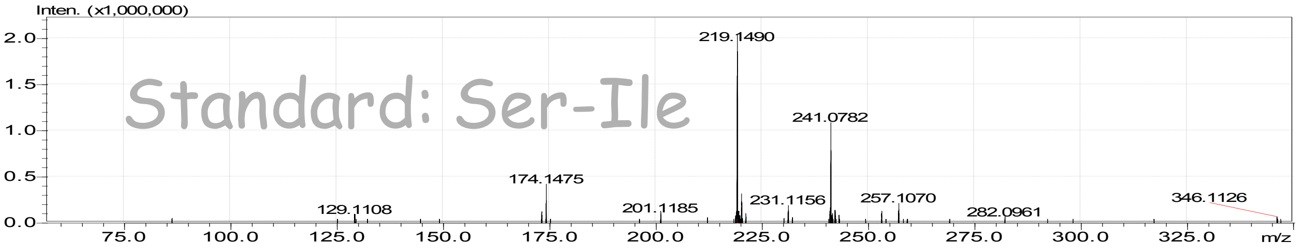
Inten. (x1,000,000)
Standard: Ser-Ile
219.1490
241.0782
174.1475
129.1108
201.1185
231.1156
257.1070
282.0961
346.1126
m/z

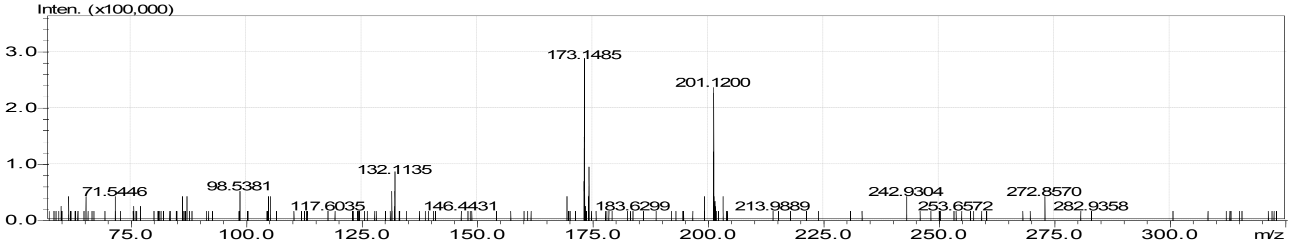
Inten. (x100,000)
173.1485
201.1200
132.1135
71.5446
98.5381
117.6035
146.4431
183.6299
213.9889
242.9304
253.6572
272.8570
282.9358
m/z

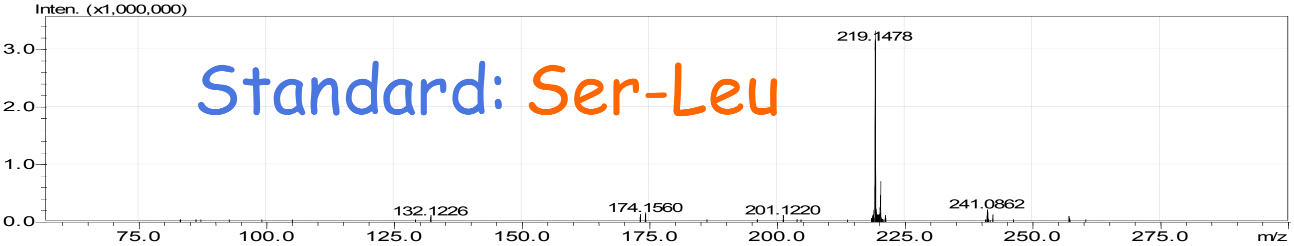
Inten. (x1,000,000)
Standard: Ser-Leu
219.1478
132.1226
174.1560
201.1220
241.0862
m/z

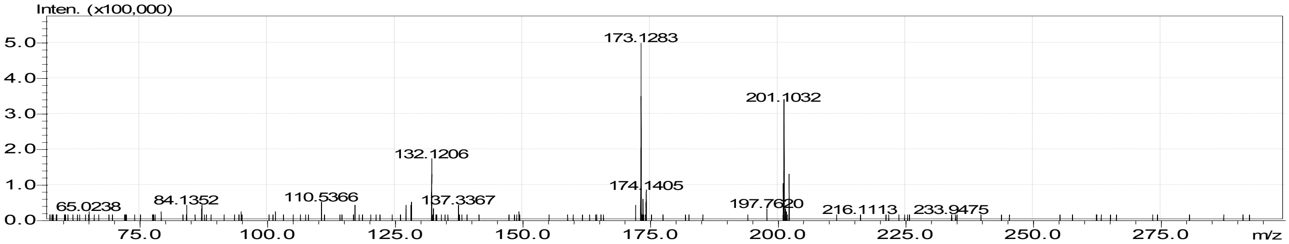
Inten. (x100,000)
173.1283
201.1032
132.1206
174.1405
65.0238
84.1352
110.5366
137.3367
197.7620
216.1113
233.9475
m/z

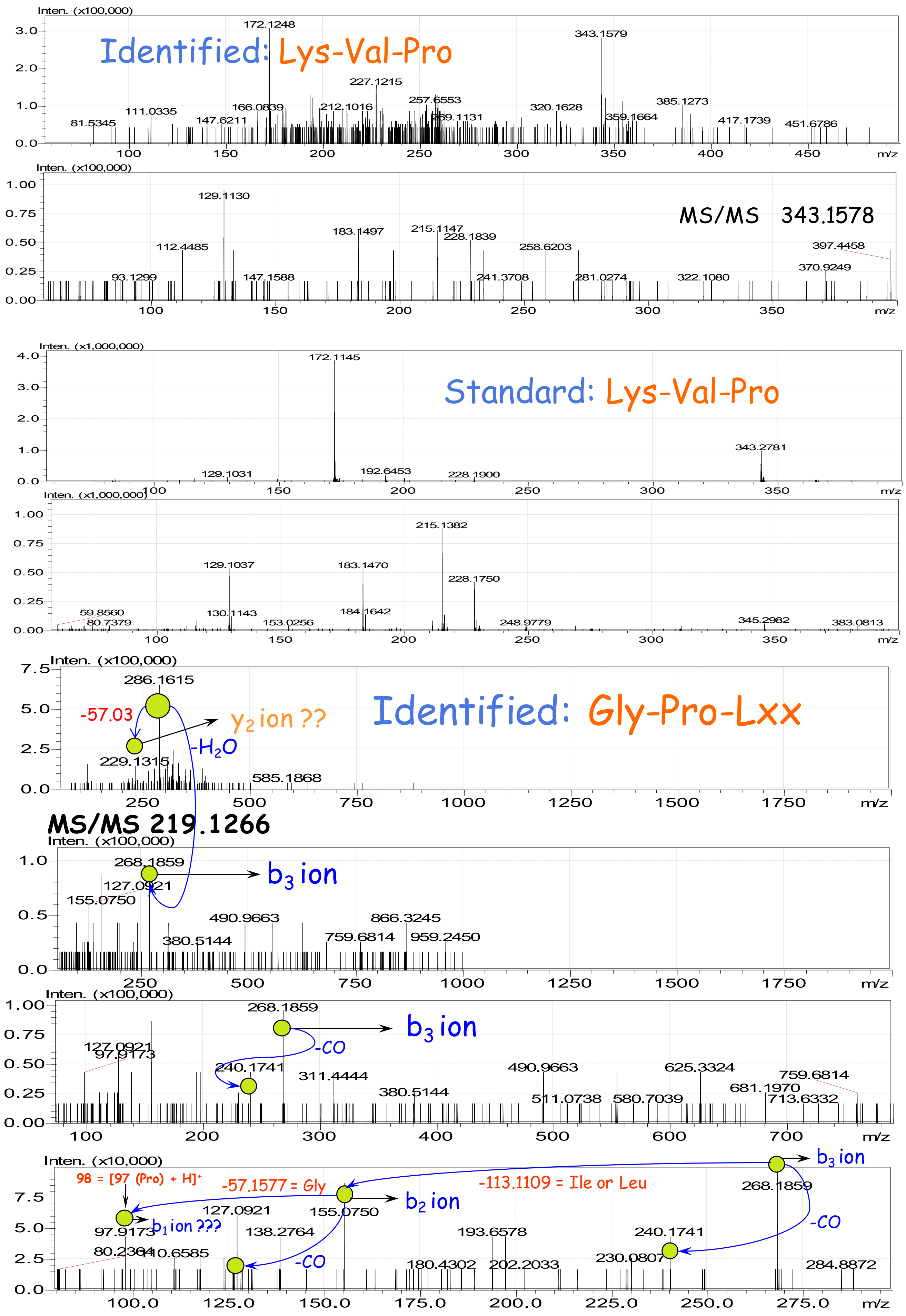

Inten. (x100,000)
Identified: Lys-Val-Pro
172.1248
343.1579
227.1215
257.6553
385.1273
166.0839
212.1016
320.1628
111.0335
269.1131
359.1664
417.1739
81.5345
147.6211
451.6786
m/z
Inten. (x100,000)
MS/MS 343.1578
129.1130
183.1497
215.1147
228.1839
112.4485
258.6203
397.4458
370.9249
93.1299
147.1588
241.3708
281.0274
322.1080
m/z
Inten. (x1,000,000)
Standard: Lys-Val-Pro
172.1145
343.2781
129.1031
192.6453
228.1900
m/z
Inten. (x1,000,000)
215.1382
129.1037
183.1470
228.1750
59.8560
80.7379
130.1143
153.0256
184.1642
248.9779
345.2982
383.0813
m/z
Inten. (x100,000)
286.1615
-57.03
y_2 ion ??
Identified: Gly-Pro-Lxx
-H_2O
229.1315
585.1868
m/z
MS/MS 219.1266
Inten. (x100,000)
268.1859
b_3 ion
127.0921
155.0750
490.9663
866.3245
759.6814
959.2450
380.5144
m/z
Inten. (x100,000)
268.1859
b_3 ion
-CO
127.0921
97.9173
240.1741
311.4444
490.9663
625.3324
759.6814
681.1970
713.6332
380.5144
511.0738
580.7039
m/z
Inten. (x10,000)
b_3 ion
98 = [97 (Pro) + H]$^+$
-57.1577 = Gly
-113.1109 = Ile or Leu
268.1859
b_2 ion
127.0921
155.0750
97.9173
b_1 ion ???
138.2764
193.6578
240.1741
-CO
80.2364
110.6585
-CO
180.4302
202.2033
230.0807
284.8872
m/z

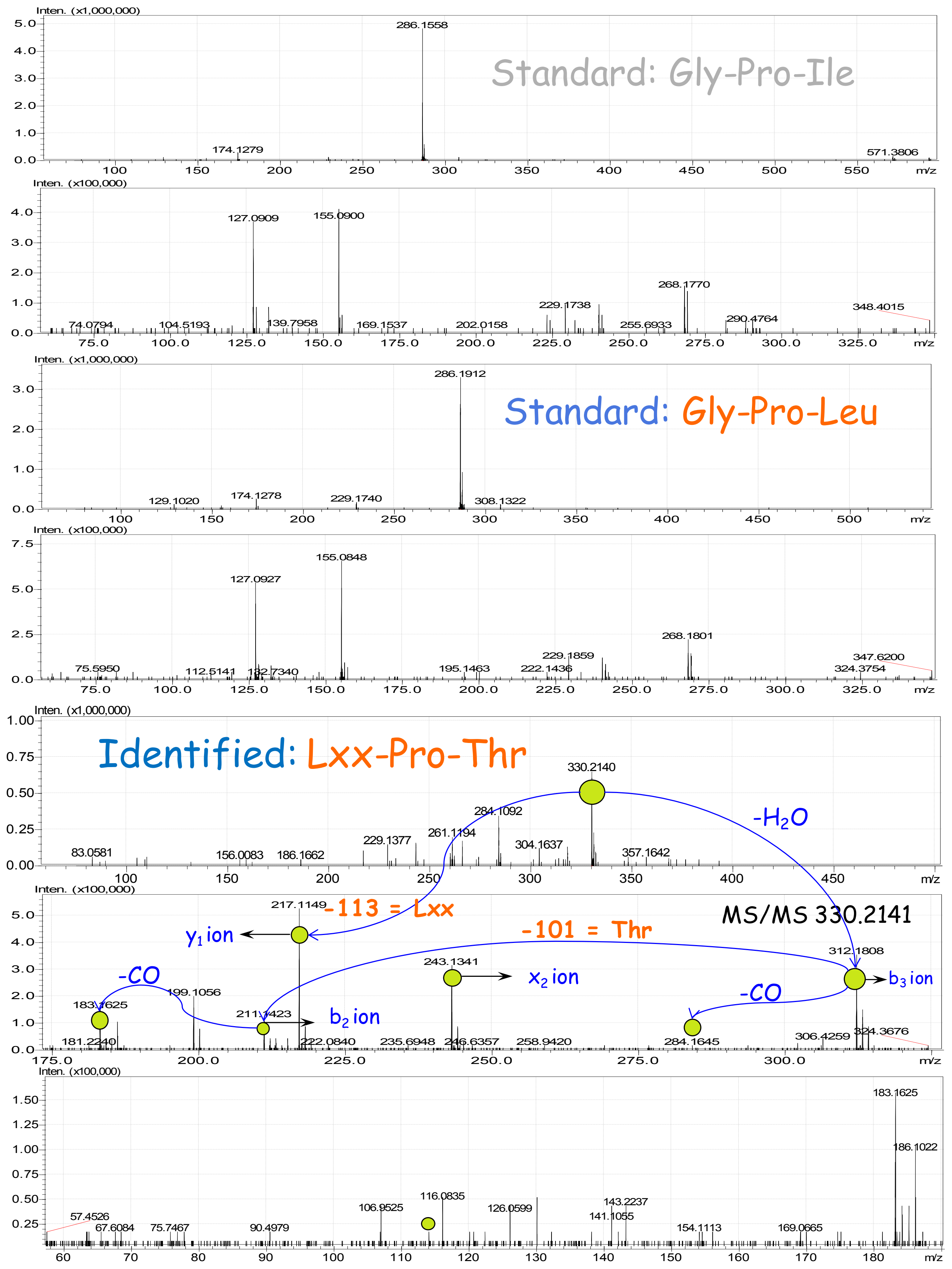
Standard: Gly-Pro-Ile
286.1558
174.1279
571.3806
127.0909
155.0900
268.1770
229.1738
348.4015
Standard: Gly-Pro-Leu
286.1912
129.1020
174.1278
229.1740
308.1322
155.0848
127.0927
268.1801
229.1859
Identified: Lxx-Pro-Thr
330.2140
284.1092
261.1194
229.1377
-H2O
MS/MS 330.2141
217.1149
-113 = Lxx
-101 = Thr
y1 ion
312.1808
b3 ion
243.1341
x2 ion
-CO
183.1625
199.1056
211.1423
b2 ion
-CO
284.1645
324.3676
183.1625
186.1022
116.0835
106.9525
126.0599
143.2237
141.1055

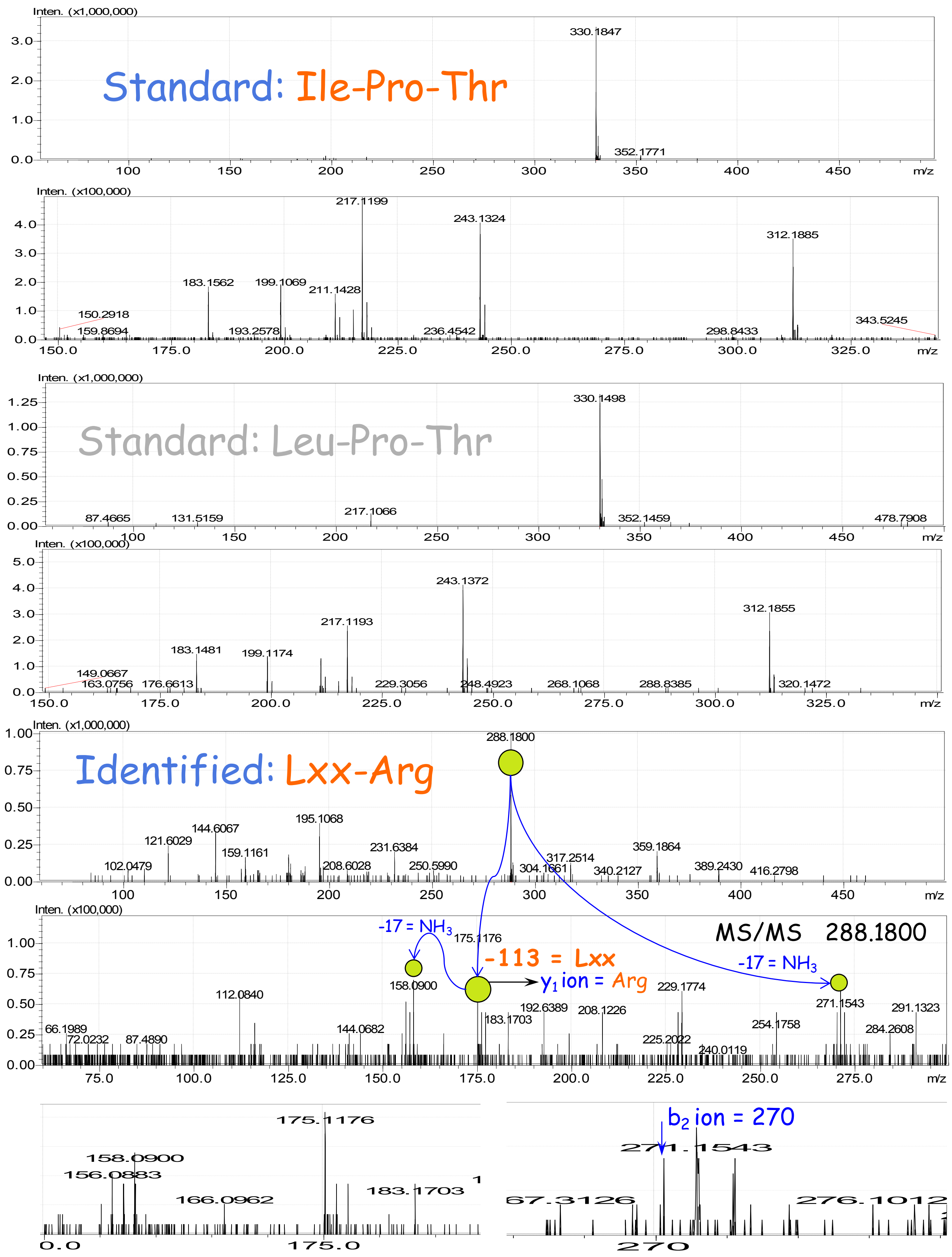

Standard: Ile-Pro-Thr
Inten. (x1,000,000)
330.1847
352.1771
Inten. (x100,000)
217.1199
243.1324
312.1885
183.1562
199.1069
211.1428
150.2918
159.8694
193.2578
236.4542
298.8433
343.5245
Standard: Leu-Pro-Thr
Inten. (x1,000,000)
330.1498
87.4665
131.5159
217.1066
352.1459
478.7908
Inten. (x100,000)
243.1372
217.1193
312.1855
183.1481
199.1174
149.0667
163.0756
176.6613
229.3056
248.4923
268.1068
288.8385
320.1472
Identified: Lxx-Arg
Inten. (x1,000,000)
288.1800
195.1068
144.6067
121.6029
159.1161
102.0479
231.6384
208.6028
250.5990
317.2514
304.1661
340.2127
359.1864
389.2430
416.2798
Inten. (x100,000)
MS/MS 288.1800
-17 = NH3
175.1176
-113 = Lxx
y1 ion = Arg
158.0900
-17 = NH3
112.0840
229.1774
271.1543
291.1323
192.6389
208.1226
183.1703
144.0682
254.1758
284.2608
66.1989
72.0232
87.4890
225.2022
240.0119
m/z
175.1176
158.0900
156.0883
166.0962
183.1703
b2 ion = 270
271.1543
67.3126
276.1012

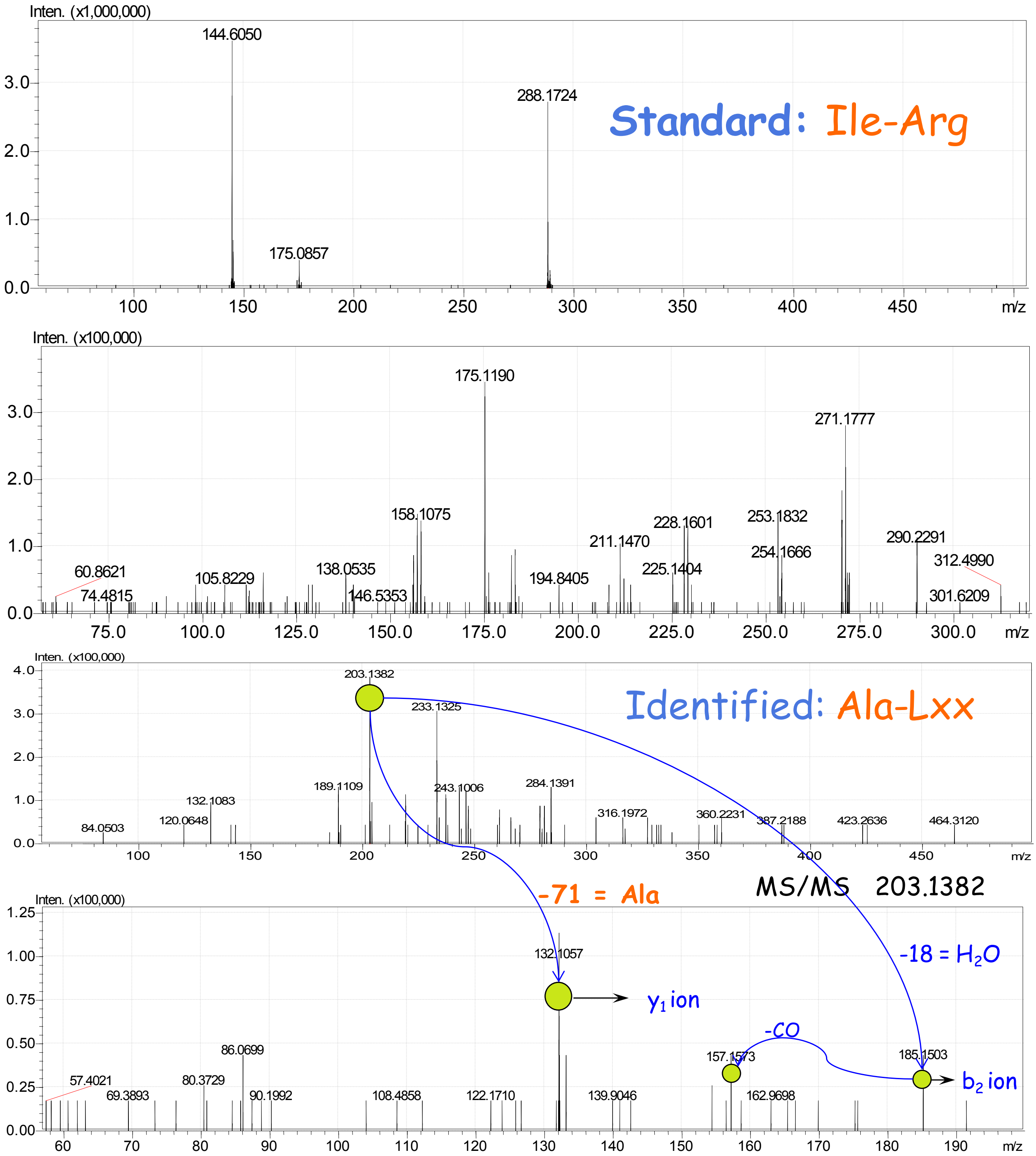
Inten. (x1,000,000)
144.6050
288.1724
175.0857
Standard: Ile-Arg
m/z
Inten. (x100,000)
175.1190
271.1777
158.1075
228.1601
253.1832
211.1470
290.2291
254.1666
312.4990
60.8621
105.8229
138.0535
194.8405
225.1404
74.4815
146.5353
301.6209
m/z
Inten. (x100,000)
203.1382
233.1325
Identified: Ala-Lxx
189.1109
243.1006
284.1391
132.1083
316.1972
360.2231
387.2188
423.2636
464.3120
120.0648
84.0503
m/z
-71 = Ala
MS/MS 203.1382
Inten. (x100,000)
132.1057
-18 = H_2O
y_1 ion
-CO
157.1573
185.1503
b_2 ion
86.0699
57.4021
80.3729
69.3893
90.1992
108.4858
122.1710
139.9046
162.9698
m/z

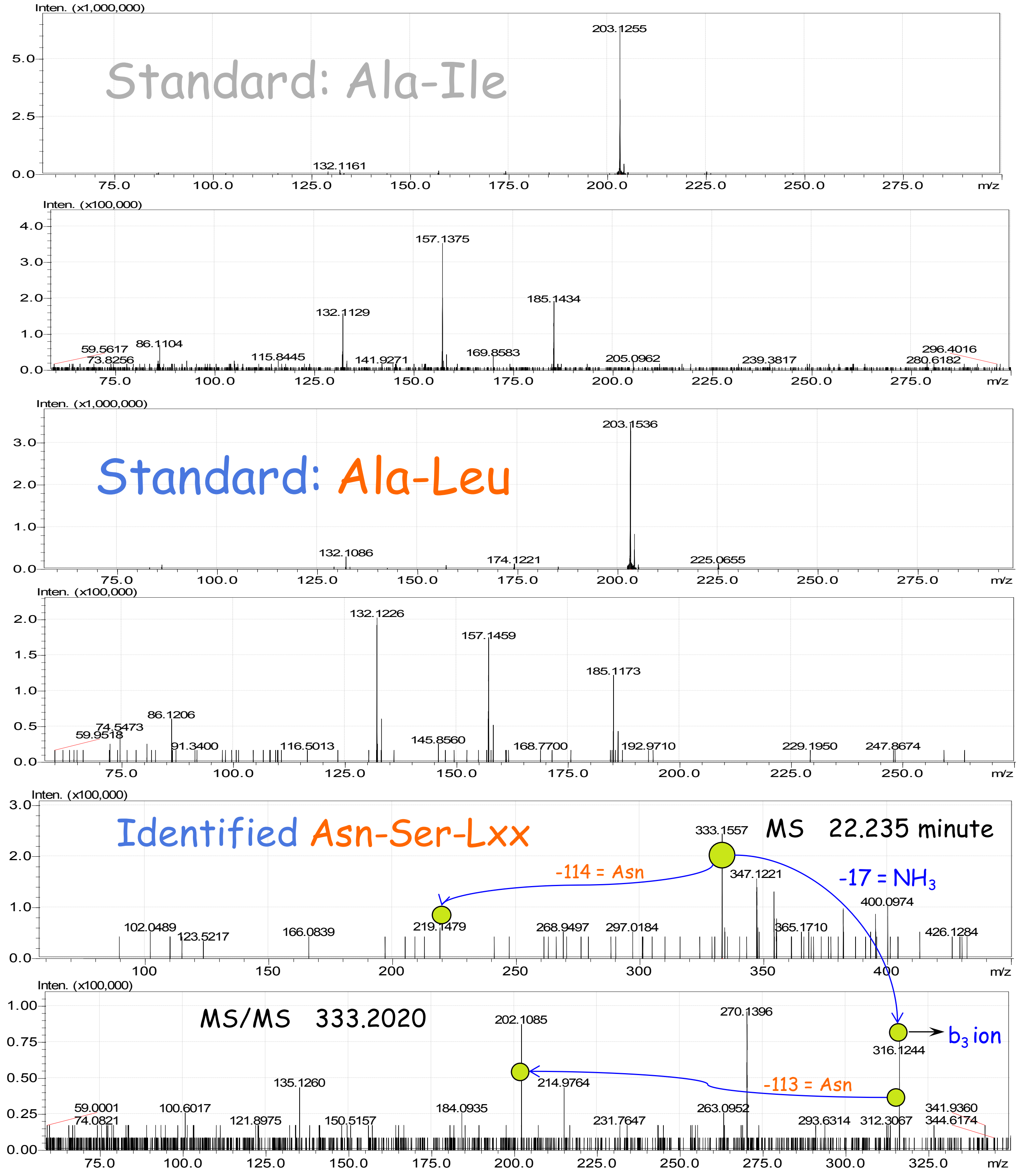

Inten. (x1,000,000)
Standard: Ala-Ile
203.1255
132.1161
m/z
Inten. (x100,000)
157.1375
185.1434
132.1129
59.5617
86.1104
73.8256
115.8445
141.9271
169.8583
205.0962
239.3817
296.4016
280.6182
Inten. (x1,000,000)
Standard: Ala-Leu
203.1536
132.1086
174.1221
225.0655
Inten. (x100,000)
132.1226
157.1459
185.1173
86.1206
74.5473
59.9518
91.3400
116.5013
145.8560
168.7700
192.9710
229.1950
247.8674
Inten. (x100,000)
Identified Asn-Ser-Lxx
333.1557
MS 22.235 minute
-114 = Asn
347.1221
-17 = NH3
400.0974
102.0489
123.5217
166.0839
219.1479
268.9497
297.0184
365.1710
426.1284
Inten. (x100,000)
MS/MS 333.2020
202.1085
270.1396
b3 ion
316.1244
135.1260
214.9764
-113 = Asn
59.0001
74.0821
100.6017
121.8975
150.5157
184.0935
231.7647
263.0952
293.6314
312.3067
341.9360
344.6174

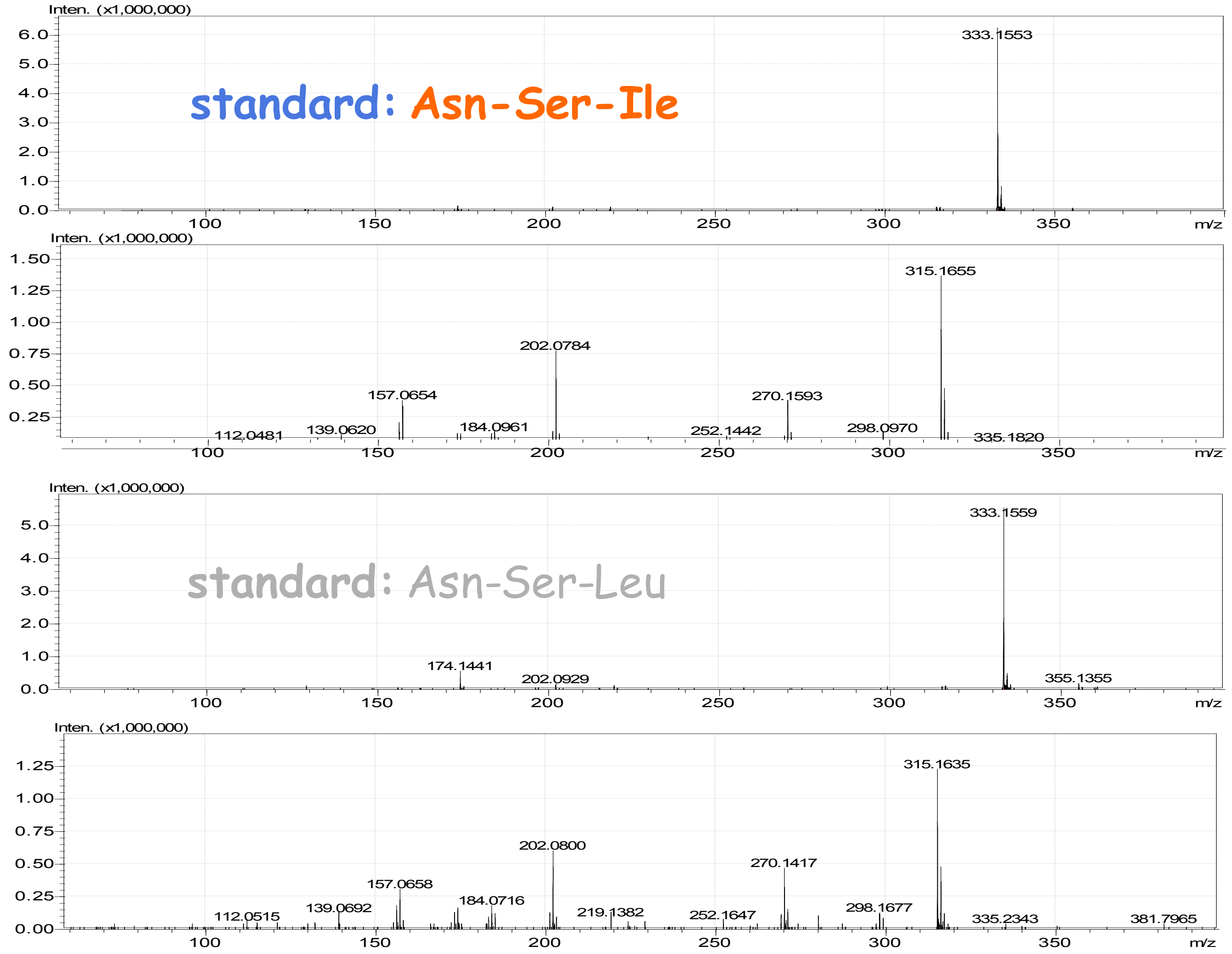
Inten. (x1,000,000)
333.1553
standard: Asn-Ser-Ile
m/z
Inten. (x1,000,000)
315.1655
202.0784
157.0654
270.1593
112.0481
139.0620
184.0961
252.1442
298.0970
335.1820
m/z
Inten. (x1,000,000)
333.1559
standard: Asn-Ser-Leu
174.1441
202.0929
355.1355
m/z
Inten. (x1,000,000)
315.1635
202.0800
270.1417
157.0658
184.0716
139.0692
112.0515
219.1382
252.1647
298.1677
335.2343
381.7965
m/z

Table S1. Eleven fractions and their ACE inhibition rates.

No.	Samples	Code	Sample Final Concentration (100 µg/mL)				
			ACE Inhibition Rate (%)			Mean	Std.
1	Hot water extract	HWR	43.33	42.12	38.04	41.16	0.87
2	Auto-digested Reishi	ADR	66.10	67.79	67.91	67.27	1.01
	time (min)-**amount (mg)**						
1	5–13 **(162.9)**	ADR1	21.04	22.50	22.94	22.16	1.00
2	13–17.5 **(48.7)**	ADR2	70.14	72.05	70.59	70.93	1.00
3	17.5–18.5 **(4.7)**	ADR3	74.29	74.51	75.41	74.74	0.59
4	18.5–22 **(14.4)**	ADR4	76.31	76.98	77.20	76.83	0.47
5	22–29.5 (**29.9**)	ADR5	88.42	89.87	90.66	**89.65**	1.14
6	29.5–31.5 **(6.45)**	ADR6	77.54	78.33	78.89	78.25	0.68
7	31.5–33.5 **(3.21)**	ADR7	83.48	83.93	84.16	83.86	0.34
8	33.5–35 **(1.8)**	ADR8	86.29	86.73	87.18	86.73	0.45
9	35–37.5 **(2.7)**	ADR9	86.51	88.30	87.07	87.29	0.92
10	37.5–39 **(1.2)**	ADR10	86.85	86.73	87.07	86.88	0.17
11	39–60 **(18.8)**	ADR11	80.23	80.90	79.67	80.27	0.62

Table S2. Sub-fractions and their ACE inhibition rates.

No.	Time (min)–Amount (mg)	Code	Sample Final Concentration (100 µg/mL)				
			ACE Inhibition Rate (%)			Mean	Std.
1	6–22.5 (3.48)	ADR5-1	85.41	83.11	83.77	84.10	1.18
2	22.5–24 (2.63)	ADR5-2	66.94	67.52	69.40	67.95	1.29
3	24–34.2 (2.48)	ADR5-3	91.16	91.16	92.80	91.71	0.95
4	34.2–35.6 (0.61)	ADR5-4	86.64	86.32	88.70	87.22	1.29
5	35.6–41.5 (4.39)	ADR5-5	80.49	81.64	81.39	81.17	0.61
6	41.5–44.5 (1.67)	ADR5-6	81.55	80.41	79.91	80.62	0.84
7	44.5–65 (5.68)	ADR5-7	88.78	88.45	89.60	88.94	0.59
